# Seamless multi-skill learning: learning and transitioning non-similar skills in quadruped robots with limited data

**DOI:** 10.3389/frobt.2025.1542692

**Published:** 2025-04-30

**Authors:** Jiaxin Tu, Peng Zhai, Yueqi Zhang, Xiaoyi Wei, Zhiyan Dong, Lihua Zhang

**Affiliations:** Academy for Engineering and Technology, Fudan University, Shanghai, China

**Keywords:** multi-skill learning, imitation learning, adaptive command sampling, self-trajectory augmentation, quadrupedal robots

## Abstract

In multi-skill imitation learning for robots, expert datasets with complete motion features are crucial for enabling robots to learn and transition between different skills. However, such datasets are often difficult to obtain. As an alternative, datasets constructed using only joint positions are more accessible, but they are incomplete and lack details, making it challenging for existing methods to effectively learn and model skill transitions. To address these challenges, this study introduces the Seamless Multi-Skill Learning (SMSL) framework. Integrated within the Adversarial Motion Priors framework and incorporating self-trajectory augmentation techniques, SMSL effectively utilizes high-quality historical experiences to guide agents in learning skills and generating smooth, natural transitions between them, addressing the learning difficulties caused by incomplete expert datasets. Additionally, the research incorporates an adaptive command sampling mechanism to balance the training opportunities for skills of various difficulties and prevent catastrophic forgetting. Our experiments highlight potential issues with baseline methods when imitating incomplete expert datasets and demonstrate the superior performance of the SMSL framework. Sim-to-real experiments on real Solo8 robots further validate the effectiveness of SMSL. Overall, this study confirms the SMSL framework’s capability in real robotic applications and underscores its potential for autonomous skill learning and generation from minimal data.

## 1 Introduction

### 1.1 Background

In the field of robotic control, Reinforcement Learning (RL) has been proven to be an effective control method, particularly in legged robots (Lee et al., 2020; Kumar et al., 2021; Miki et al., 2022; Hou et al., 2024). Despite these successes, robots need to acquire complex skills and dynamically switch tasks to cope with fluctuating environments, presenting significant challenges to RL methods that rely on intricate reward designs. Imitation Learning (IL) enables robots to learn from expert datasets without the necessity for complex reward functions, thus allowing platforms such as quadruped robots to master a variety of motion skills (Li et al., 2023). However, IL is heavily dependent on the quality and completeness of the expert datasets (Ran and Su, 2024). When the expert datasets are of high quality, the integration of Generative Adversarial Networks (GAN) (Goodfellow et al., 2020) with RL (Sutton and Barto, 2018) in the Adversarial Motion Priors (AMP) (Peng et al., 2021) approach demonstrates its superiority. By training discriminators to differentiate between expert data and policy outputs, AMP encourages policy networks to produce similar state transitions, performing well on high-quality datasets. These datasets typically include physical information such as joint positions, velocities, accelerations, and torques, which are crucial for enhancing the effectiveness of IL.

Ideally, these datasets are obtained through high-precision motion capture equipment, but such devices may fail to capture all high-speed or complex movements. As an alternative, researchers generate high-quality expert data using trajectory optimization algorithms or deep RL methods in simulation environments. These approaches undoubtedly increase the initial preparation costs for IL methods. A simpler and less costly method involves acquiring joint position information from video recordings or by manually manipulating real robots. However, its effectiveness is reduced due to the lack of rich motion feature information. Moreover, when expert datasets contain skills of various complexities, the AMP method struggles to train a policy that learns all the skills in the dataset; it may learn some skills but also risk forgetting previously learned ones over extended training periods. It is more likely to primarily learn simple or mixed skills, which may not meet researchers’ expectations. To optimize the AMP approach, one potential method is to manually segregate various strategies within the expert dataset and employ separate networks to learn them, but this method requires extensive manual annotation and computational resources. When the skills to be imitated differ significantly in terms of similarity, they are referred to as Non-similar skills in this context. For example, in a quadruped robot, transitioning between quadrupedal and bipedal states is considered a Non-similar skill transition. If the expert dataset only contains these states without the transitions between them, simple IL methods become less effective in achieving the research objectives (Hussein et al., 2017). The challenge is more pronounced in scenarios requiring transitions between fundamentally different motion skills, making it difficult to effectively learn and transition between skills.

**FIGURE 1:**
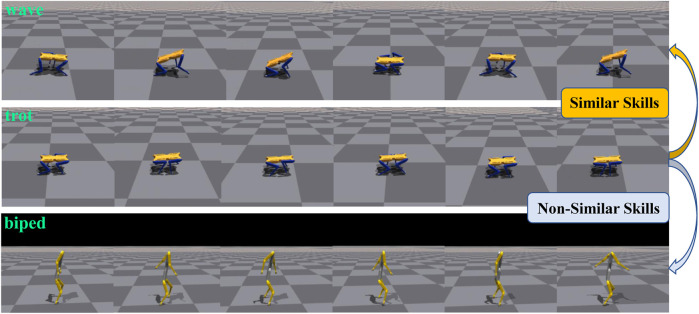
Simulation description of Similar skills and Non-similar skills. Three motor skills of“ wave” (Top),“ trot” (Mid) and “biped” (Low) are selected to show the characteristics of Similar skills and Non-similar skills.

**FIGURE 2:**
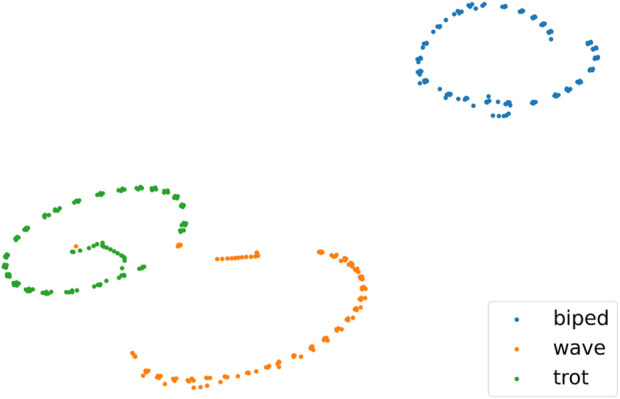
State trajectory distribution diagrams for Similar and Non-similar skills: Shows the state trajectory distribution of Similar and Non-similar skills, including joint position information for “wave”, “trot”, and “biped”.

Non-similar skills refer to those with significant differences in motion characteristics. We employed simulation, t-SNE, and Dynamic Time Warping (DTW) techniques (Müller, 2007) to visually depict the differences between motion skills. In Figure 1, we selected three skills simulated using the Isaac Gym platform to demonstrate the differences in motion characteristics between Similar and Non-similar skills. ‘wave’ and ‘trot’, both based on a quadrupedal stance, are categorized as similar skills. Conversely, ‘trot’ and ‘biped’, based on quadrupedal and bipedal stances respectively, are considered Non-similar skills. Figure 2 presents t-SNE plots that display the state trajectories of two skills (‘wave’, ‘trot’) from the Cassie expert dataset, compared with the state trajectories from our Non-similar expert dataset (‘biped’), where the trajectories include joint position information. It can be observed that the trajectories of Similar skills (‘wave’, ‘trot’) exhibit some overlap in the state space, providing favorable conditions for designing strategies capable of mastering multiple skills. However, the Non-similar skill (‘biped’) does not overlap with the other skills, which increases the training complexity of multi-skill strategies. We also used DTW to quantify the distances between Similar and Non-similar skills, as shown in Table 1. We calculated the distances between the state trajectories of different skills, with smaller values indicating greater similarity between skills. The gaps between Non-similar skills are significantly larger than those between Similar skills.

**TABLE 1:** Similarity of Joint Position States in Similar Skills and Non-similar skills (DTW).

Skills	Wave	Biped
trot	16.14	**38.22**

Given these challenges, effectively learning distinct skills and transitioning from an expert dataset containing only joint positions has emerged as a critical research issue. This involves one of the core issues in RL: Avoid catastrophic forgetting. To address this issue, we focus on the following two key questions in this research: (1) How to ensure that both complex and simple Non-similar skills receive appropriate training opportunities within a unified policy network, thus achieving comprehensive skill mastery? (2) How to compensate for missing states between skills within the same policy network to achieve more accurate and effective skill switching, thereby improving policy performance and ensuring seamless transitions between different actions?

### 1.2 Contributions

**FIGURE 3:**
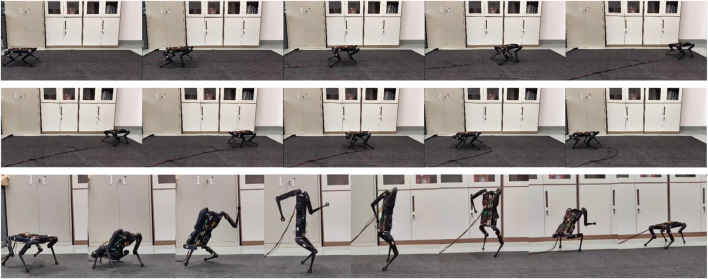
Sim-to-real task is conducted on the Solo8 robot, involving moving forward (Top) and backward (Mid) in a quadrupedal walking state, and transitioning from a quadrupedal state to a bipedal walking state (Low). Our approach uses a single policy to manage the transition between quadrupedal and bipedal states.

To address these problems above, this paper introduces Seamless Multi-Skill Learning (SMSL), a method for learning multi-non-similar skills with limited data. This approach incorporates an adaptive skill selection mechanism to deal with expert datasets with Non-similar skills expert datasets. This selection mechanism ensures that skills of various difficulties can be sufficiently trained and achieve comprehensive mastery of the skills. It also effectively extracts skills and generates natural transitions between Non-similar skills by leveraging historically successful states. The contributions of this method can be summarized as follows:

• We introduce a novel adaptive skill selection method that samples skill commands based on the learning progress of each skill, balancing skill acquisition and preventing catastrophic forgetting;

• We have integrated an experience replay module into the AMP framework. This module dynamically utilizes historical successful states from the training process as the foundation for environmental initialization, compensating for the lack of information in the expert dataset, and facilitating the imitation learning of Non-similar skills and the generation of transition actions;

• Our method has been validated on both a simulation platform and the real-world Solo8 robot platform (Figure 3), which outperforms baseline algorithms. Ablation studies demonstrate the effectiveness of our proposed method.

## 2 Related work

Quadruped robots enhance the flexibility and efficiency of task execution by mastering multiple skills to cope with the complexity of task environments (Aziz et al., 2022). Due to the complexity of controlling quadruped robots, traditional RL methods encounter challenges related to the complexity and potential imprecision of reward design in multi-skill tasks. As an efficient alternative, IL enables quadruped robots to quickly master complex skills by observing and replicating expert behaviors, making it particularly suitable for complex tasks that require the rapid integration of multiple skills (Billard et al., 2008; Torabi et al., 2019; Gavenski et al., 2024; Oh et al., 2018).

Imitation learning has seen a series of innovative results in recent research on quadruped robot motion skills. Generative Adversarial Imitation Learning (GAIL) (Ho and Ermon, 2016) offers an effective framework for imitation learning in high-dimensional environments by learning from expert data containing state-action pairs without relying on explicit cost functions. AMP (Peng et al., 2021) extends this by guiding robots to complete tasks using expert data and the task environment, even with only state trajectory data. Based on AMP, (Escontrela et al., 2022; Wu et al., 2023), applied the AMP method for robust and agile quadruped walking on complex terrains. (Vollenweider et al., 2023). developed a Multi-AMP structure for multi-skill strategies, pairing each expert dataset with its own generative adversarial network. However, generative adversarial networks can experience mode collapse when handling numerous, unlabeled datasets and skills. To address this, Cassi (Li et al., 2023) introduced a more effective approach integrating generative adversarial networks and unsupervised skill discovery techniques, enabling policy to imitate skills from expert data and maximizing mutual information between skills and a latent variable z . While effective with unlabeled expert datasets, these strategies’ efficacy depends on dataset quality and completeness. As discussed in Section 1.1, the learning performance can be adversely affected when the expert dataset is incomplete. To address this, we introduce a method for reusing historical experiences to reinforce skill learning within the strategy. Additionally, we have designed an adaptive skill selection method that selects the next skill by calculating the rewards generated for different skills during training, thus providing more learning opportunities for poorly performing skills.

Experience replay is an extremely efficient strategy guidance technique in RL. It enables robots to learn under conditions of limited samples, thereby enhancing policy learning efficiency (Yang et al., 2024). This technique balances exploration and exploitation, avoiding ineffective trial-and-error processes and catastrophic forgetting (Lin, 1992; Mnih et al., 2015; Schaul et al., 2015). Studies like (Peng et al., 2018; Li et al., 2023) introduce expert datasets into the experience replay buffer to initialize robots. During initialization, the robots’ state may be sampled from the expert dataset or generated randomly. However, these methods depend on the expert datasets’ prior knowledge. Limited or non-diverse datasets can hinder the strategies’ adaptability to various states in complex environments (Rajaraman et al., 2020). This dependency weakens the generalization of the policy in new environments and may lead robots to replicate errors or suboptimal behaviors, limiting the effectiveness of the policy and the robot’s adaptability and robustness (Cao et al., 2024; Lan et al., 2023) introduces the Self-Trajectory Augmentation (STA) technique, which dynamically collects and integrates excellent historical trajectories generated by the robot during training. This addresses the aforementioned issues by expanding the diversity and coverage of the dataset. (Messikommer et al., 2024). demonstrates that strategically selecting and utilizing past experienced states to initialize robots enhances performance in complex tasks. To our knowledge, our method introduces the STA approach within the AMP framework for the first time, reusing excellent historical states to effectively avoid catastrophic forgetting in multi-skill learning, thereby enhancing the agent’s learning process.

## 3 Preliminaries

In this work, the environment is modeled as an infinite-horizon Markov decision process (MDP), defined by the tuple $M=\left(S,O,A,p,r_{t},p_{0},\gamma \right)$ . S represents the state space, which includes base linear velocity v , base angular velocity ω , base quaternion (x,y,z,w) , base height h , joint positions p , joint velocities $\dot{p}$ , as well as the action a from the previous moment. O represents the observations from the real world, serving as input for the policy network. It includes all values in the state space S except the base linear velocity v , base angular velocity ω , and footstep positions. A is the action space that indicates the changes in joint positions. $p=\left(s^{\prime }|s,a\right)$ is the transition dynamics, $r_{t}\left(s,a,s^{\prime }\right)$ is the reward function, $p_{0}$ is the initial state distribution, and γ∈[0,1) is the discount factor. The objective of RL is to find the optimal parameters θ of a policy $\pi_{\theta }:S\mapsto A$ that maximize the expected discounted reward $J\left(\theta \right)=E_{\pi_{\theta }}\left[\sum_{t}\gamma^{t}r_{t}\right]$ .

### 3.1 Adversarial Motion Priors framework

Currently, frameworks that integrate GAN and RL methods are widely used in the field of IL. We have opted to use the AMP framework to emulate the general motion characteristics found in these expert datasets. Similarly to Escontrela et al. (2022), the imitation discriminator $d_{\psi }$ is optimized using the least squares GAN (LSGAN) method by estimating scores of +1 for state transitions from the expert dataset and −1 for those of the policy. The discriminator’s objective function (Equation 1) is represented as follows:

$$E_{d^{M}}\left[{\left(d_{\psi }\left(o^{\text{I}}\right)-1\right)}^{2}\right]+E_{d^{\pi }}\left[{\left(d_{\psi }\left(s^{\psi }\right)+1\right)}^{2}\right]+w^{\text{GP}}E_{d^{M}}\left[\|\nabla_{{\text{o}}^{\text{I}}}d_{\psi }\left(o^{\text{I}}\right){\|}_{2}^{2}\right],$$ (1)

where the last term represents the non-zero gradient penalty for state transitions from the expert dataset, with $w^{\text{GP}}$ as the weight parameter. The purpose of the imitation discriminator is to distinguish between the state transition distribution $d^{M}$ from the expert dataset and the state transition distribution $d^{\pi }$ generated by the policy. Random state transitions are sampled from the expert dataset as $o^{\text{I}}=\left[\left(o_{t-1}^{\text{I}},o_{t}^{\text{I}}\right){|}_{t=\left(1,2,3,\ldots \right)}\right]$ . It is important to note that the dataset does not contain actions for transitioning between motor skills, and any sudden changes between skills are considered negligible. We use $s^{\psi }=\left[\left(s_{t-1}^{\psi },s_{t}^{\psi }\right){|}_{t=\left(1,2,3,\ldots \right)}\right]$ as the output of state transitions by the policy. In both $o_{t}^{\text{I}}$ and $s_{t}^{\psi }$ , only the position information of the robot’s joints is included. The discriminator aids in policy optimization through the following reward function:

$$r^{\text{I}}=\max\left[0,1-0.25{\left(d_{\psi }\left(s^{\psi }\right)-1\right)}^{2}\right].$$ (2)

## 4 Methods

### 4.1 Overview

**FIGURE 4:**
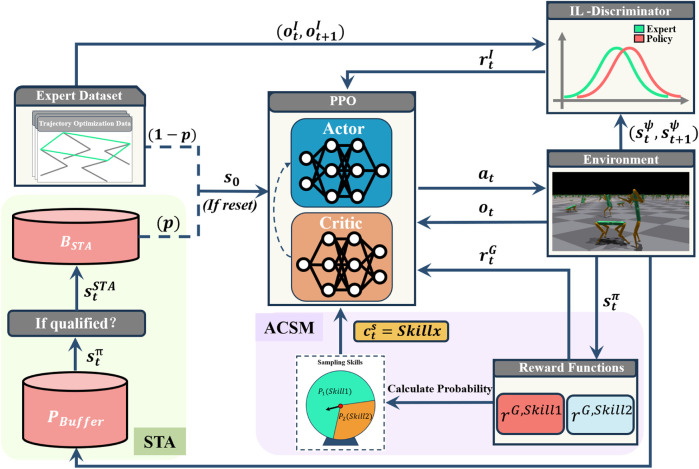
Method Overview. The system consists of two key components that work collaboratively to facilitate efficient skill learning: Adaptive Command Selection Mechanism (Purple) and Self-Trajectory Augmentation module (Green). Adaptive Command Selection module intelligently selects the optimal command by weighing the rewards of different skills, thereby balancing the opportunities for skill training. $r^{G,Skill1}$ and $r^{G,Skill2}$ represent the goal rewards obtained for different skills, respectively. $P_{1}$ and $P_{2}$ represent the sampling probabilities of different skills, and before each sampling, the probabilities are recalculated based on their corresponding goal reward values. Self-Trajectory Augmentation module selects optimal states from $P_{\mathrm{Buffer}}$ based on quality criteria, then adds them to $B_{\mathrm{STA}}$ , and samples from $B_{\mathrm{STA}}$ with probability p during initialization.

Our goal is to learn various motion skills from an expert data set that lacks complete information and to achieve transitions between these skills at any moment. To achieve adaptive training frequencies for skills of varying difficulty, we have designed a command selector denoted as C , which includes both velocity commands and motion skill commands, mathematically expressed as $C=\left[c^{v},c^{s}\right]$ . Here, $c^{v}\in \left[-0.5,0.5\right]$ is the velocity command and $c^{s}\in \left\{\left\{1,0\right\},\left\{0,1\right\}\right\}$ are the motion skill commands, with time t omitted for simplicity. Our research uses a quadruped robot capable of both bipedal and quadrupedal walking to validate the performance of our methods. Specifically, {1,0} indicates the command for bipedal walking, while {0,1} indicates the command for quadrupedal walking. Our reward function (Equations 3, 4) is structured as follows:

$$r_{t}=w^{I}r_{t}^{I}+w^{G}r_{t}^{G},$$ (3)

$$r_{t}^{G}=r_{t}^{\text{Q}}+r_{t}^{\text{B}},$$ (4)

where $r_{t}^{\text{Q}}$ is the goal reward for the quadrupedal state, and $r_{t}^{\text{B}}$ is the goal reward for the bipedal state, which will be introduced in Section 4.4. Furthermore, we introduce the STA method, maintaining an initialized STA buffer that stores favorable states acquired during the training process. This buffer allows for probability sampling p∈[0,1] when resetting the environment, facilitating policy learning. Both $w^{I}$ and $w^{G}$ are weight coefficients. Figure 4 provides an overview of the schematic diagram of our method. In this study, the expert dataset comprises only joint position variation data for locomotion skills and the base’s quaternion, lacking data on transitions between different skills. During training, at the start of each episode, the system samples the initial pose from the expert dataset with a probability of (1−p) and from the self-trajectory augmented buffer $\left(B_{\mathrm{STA}}\right)$ with a probability of p . The green area in the figure delineates the STA module, which is responsible for selecting high-quality robot states from historical data and storing them in $B_{\mathrm{STA}}$ . The buffer $B_{\mathrm{STA}}$ includes joint position information, quaternions, joint velocities, and can be extended according to task requirements. Subsequently, the agent interacts with the environment, obtaining states $s_{t}^{\pi }\in S$ and computing the goal reward $r_{t}^{G}$ . By inputting the joint position variations from the state transitions into the IL discriminator, an imitation reward $r_{t}^{I}$ is generated. Following this, the ACSM module, highlighted in the purple area, calculates the sampling probabilities for each skill based on their respective goal rewards, thereby adaptively selecting the skill to be trained. Finally, the policy network and the value network are updated using the PPO algorithm.

### 4.2 Adaptive Command Selection Mechanism (ACSM)

During the training process for quadruped robots, four-legged walking postures often receive higher rewards, while bipedal standing postures tend to result in frequent falls in the initial stages of training due to the agent’s difficulty in maintaining balance, leading to lower rewards. Without constraints, the agent may tend to learn those simpler, higher-reward skills. Therefore, in the training process of multi-skill strategies, it is crucial to balance the training among skills of different difficulty levels to ensure that each skill reaches a satisfactory performance level. Thus, we aim to implement a control mechanism that allocates training resources based on the performance of each skill, ensuring a balanced development of skill training.

In RL, the goal reward value at each timestep, $r_{t}^{G}$ , is naturally a crucial metric for assessing the agent’s performance. When a robot performs actions that align with human expectations, it receives higher rewards. In our method, for transitions between bipedal and quadrupedal states, we use a one-hot encoding method for differentiation. Therefore, using the encoding of commands to distinguish the environments corresponding to different commands is a natural choice when calculating the goal rewards. We use the following Equation 5 to calculate the reward ratio for each command:

$${\bar{r}}_{t}^{G,c^{s}}=\text{mean}\left(\underset{N_{x}}{\sum }\left(r_{t}^{G,c^{s}}\cdot I\left(c^{s}=x\right)\right)\right),$$ (5)

where ${\bar{r}}_{t}^{G,c^{s}}$ represents the average reward obtained in the environment under command $c^{s}$ , and $N_{x}$ represents the number of commands. Furthermore, since the task reward function is designed manually, we can estimate the reward values for the optimal states $r^{G,c^{s*}}$ . Thus, an evaluation metric for assessing the quality of skill training under each command can be obtained (Equation 6):

$$p\left(c^{s}=x\right)=\frac{{\bar{r}}_{t}^{G,c^{s}}}{r^{G,c^{s^{*}}}}.$$ (6)

Consequently, the performance evaluation metric p under different skill commands is used to determine which skill to train in the next scenario. The greater $p\left(c^{s}=x\right)$ is, the smaller the probability that the corresponding skill command $c^{s}$ will be sampled, thereby achieving the objective of adaptive training across multiple skills. In the experiment, we set the duration of each scenario to 1,000 timesteps to ensure smooth transitions between skills. Accordingly, we have designed the duration for each skill to be 500 timesteps.

### 4.3 Self-trajectory Augmentation (STA)

When training for transitions between Non-similar skills, we face a challenge: the lack of transitional actions between skills in the datasets. To enable the agent to smoothly transition from the current skill to the target skill upon receiving a skill-switch command, we propose the addition of new states to guide the agent toward the target skill transition. Drawing on the concept of the STA method, a new buffer $\left(B_{\text{STA}}\right)$ is created that utilizes valuable historical trajectories to enhance learning efficiency. This is primarily aimed at allowing the robot to more frequently experience states conducive to transitions between skills, rather than blindly exploring new states or merely remaining in fixed states of the target skill. This approach is expected to optimize the learning process of the robot, making it more efficient and natural during skill transitions.

A good state is one that allows for large rewards to be obtained in subsequent states. Therefore, we not only value the immediate rewards for the agent but also the long-term value of the state. We use the weighted average value $\left(\mu_{t+1}\right)$ of the output from the critic network $\left(V\left(s_{t+1}\right)\right)$ and the goal reward from the previous moment $\left(r_{t}^{G}\right)$ as the criterion for assessing the quality of the state $s_{t}$ at each environment at time t+1 . This results in the following formula (Equations 7, 8), which serves as the threshold for filtering good states:

$$\mu_{t+1}^{n}=r_{t,n}^{G}+w^{\text{STA}}\gamma V_{n}\left(s_{t+1}\right),$$ (7)

$$\mu_{t+1}^{i,mean}=k\frac{\sum_{n=1}^{N}\mu_{t+1}^{n}}{N},$$ (8)

where N represents the total number of environments, and n is one of them. From the historical state collections of each environment, states where $\mu_{t+1}$ exceeds the average $\mu_{t+1}^{i,\text{mean}}$ are selected to be added to $B_{\mathrm{STA}}$ , which includes the agent’s base position, quaternion, and the positions and velocities of each joint. Clearly, by employing the STA method, it is possible to capture motion characteristics not present in suboptimal expert datasets, such as velocity information, from good historical experiences. $w^{\text{STA}}$ is weight coefficient and k is the scaling factor, respectively.

### 4.4 Goal reward function

Although imitation reward in Equation 2 offers advantages in simplifying the policy learning process, the GAN (LSGAN) method is not sensitive to expert trajectories and their surrounding state space areas Li et al. (2022). Therefore, relying solely on it as the only reward for skill learning does not accurately learn skills. For example, when training robots to perform quadrupedal and bipedal walking, relying on imitation rewards, especially excessive imitation rewards, may lead the agents to learn an unintended mix of actions. This indicates that when training with incomplete expert datasets, to avoid falling into local optima and to enable more targeted exploration, it is necessary to incorporate additional reward mechanisms to guide the learning of the policy, ensuring that agents can learn more accurate and natural behavior patterns.

#### 4.4.1 Quadrupedal motion state

##### 4.4.1.1 Quadruped gait reward function

When a quadruped robot learns to move forward or backward, it can easily fall into a suboptimal pattern of hopping, which is not the desired method of movement. We want the robot’s gait to mimic that of real quadruped animals, using an alternating gait for progression. Naturally, this leads to the consideration of imposing constraints on the robot’s gait. The design of the quadrupedal movement’s gait is as follows:

Gait one:[True,False,True,False],

Gait two:[False,True,True,False],

where True and False indicate whether the robot’s feet are touching the ground, determined by the presence of contact forces with the ground. In the gait sequence, the order of the feet [FL,FR,HL,HR] corresponds to the left front, right front, left hind, and right hind foot, respectively. We record the duration of each gait $t^{g}$ , and when transitioning to a different gait, we calculate the duration as the reward value for the gait. The maximum set duration for each gait is $t^{g*}$ . Note that the reward is calculated only when experiencing transition to a different gait. This method ensures the switching between different gaits, encouraging the robot to actively use its gait. However, it can also lead to a suboptimal situation where there is a significant disparity in the duration of two gaits. For this purpose, within the same environment, we record the duration of each gait at every timestep. The difference between the current gait’s time and the previous gait’s time is used as a penalty for gait asymmetry $t^{e}$ . The gait reward for the robot’s quadrupedal motion state is calculated using the following Equation 9:

$$r^{\text{Q\_gait}}=\omega^{\text{Q\_gait}}t^{\text{Q\_gait}}-t^{e},$$ (9)

where $\omega^{\text{Q\_gait}}$ is weight coefficient.

##### 4.4.1.2 Quadruped leg-lifting reward function

To encourage the agent to lift its feet, the Isaac Gym simulation environment provides the foot elevation $h_{\mathrm{foot}}$ at each time step. A desired foot elevation $h_{\mathrm{foot}}*=0.03cm$ is defined and the following Equation 10 is used as a reward function to promote foot lifting in the quadrupedal state:

$$r^{\text{fh}}=\omega^{\text{fh}}\exp^{-\frac{\left|h_{\text{foot}}-h_{\text{foot}}^{*}\right|}{\sigma^{\text{fh}}}},$$ (10)

where $\omega^{\text{fh}}$ is hyperparameter and $\sigma^{\text{fh}}$ is the scaling factor.

##### 4.4.1.3 Quadruped velocity tracking reward function

To encourage the agent to track velocity, the following reward function (Equation 11) is used:

$$r^{\text{Q\_v}}=\omega^{\text{v}}\exp^{-\frac{\left|c_{t}^{\text{v}}-v_{t}\right|}{\sigma^{\text{v}}}},$$ (11)

where $v_{t}$ represents the velocity of the agent’s base along the x-axis at time t . $c_{t}^{\text{v}}$ denotes the desired speed sampled from the command. $\omega^{\text{v}}$ is hyperparameter and $\sigma^{\text{v}}$ is the scaling factor.

Based on the above, the total goal reward for the quadrupedal state can be summarized as follows (Equation 12):

$$r^{\text{Q}}=r^{\text{Q\_gait}}+r^{\text{fh}}+r^{\text{Q\_v}},$$ (12)

#### 4.4.2 Bipedal motion state

Due to the absence of expert datasets for the transition from quadrupedal to bipedal motion states, it is necessary to incorporate a reward function that facilitates the transition to a bipedal state, compensating for the sparsity of the rewards during the skill switch.

##### 4.4.2.1 Bipedal gait reward function

The reward function for gait transition in the bipedal state is identical to that of the quadrupedal state, so the expression will be omitted. Gait design is as follows:

Gait one:[False,False,True,False],

Gait two:[False,False,False,True].

The sequence of foot placement in the gait is the same as in the quadrupedal state. Unlike the quadrupedal state, the bipedal gait transition is effective only when the robot is capable of maintaining a stable bipedal stance. To adjust the reward value and encourage the robot to maintain a standing posture and gait, we assess the robot’s base pitch angle and monitor the foot contact states and heights during simulations. The formula is designed as follows (Equations 13, 14):

$$r^{\text{ffh}}=0.1\cdot I\left(h\geq 0.02\right)+0.3\cdot I\left(h\geq 0.08\right)+0.6\cdot I\left(h\geq 0.16\right)+1.0\cdot I\left(h\geq 0.25\right)\cdot \lambda^{\text{pitch}},$$ (13)

$$\lambda^{\text{pitch}}=\exp^{-\frac{\sqrt{{\left(\theta^{\text{pitch}}-4.71\right)}^{2}}}{\sigma^{\text{pitch}}}},$$ (14)

where $\lambda^{\text{pitch}}$ is a threshold calculated from the current pitch angle and the target pitch angle. h represents the lifting height of the two front feet, and the indicator function I(⋅) is one only if both feet meet the threshold height, otherwise it is 0. The current pitch angle $\theta^{\text{pitch}}$ , expressed in radians, can be obtained from the Isaac Gym simulation environment. The target pitch angle for the standing posture is set to 4.71 radians. The maximum lifting threshold reward is multiplied by $\lambda^{\text{pitch}}$ to guide the robot towards achieving the target pitch angle. $\sigma^{\text{pitch}}$ is the scaling factor.

From this, we can derive the following Equation 15:

$$r^{\text{B\_gait}}=\omega^{\text{B\_gait}}\left(r^{\text{B\_gait}}+r^{\text{ffh}}\right),$$ (15)

where $\omega^{\text{B\_gait}}$ is hyperparameter.

##### 4.4.2.2 Bipedal velocity tracking reward function

This reward function (Equation 16) is similar to the quadrupedal state’s velocity tracking, but differs in that the bipedal state only tracks forward direction velocity:

$$r^{\text{B\_v}}=\omega^{\text{v}}\exp^{-\frac{\left|-\left|c_{t}^{v}\right|-v_{t}\right|}{\sigma^{v}}}.$$ (16)

##### 4.4.2.3 Bipedal base reward function

The purpose of this reward function is to make the base height of the bipedal robot as close to the set target height as possible. This reward function (Equation 17) is similar to the quadrupedal state’s velocity tracking, but differs in that the bipedal state only tracks forward direction velocity:

$$r^{\text{B\_base}}=\omega^{\text{B\_base}}\exp^{-\frac{\sqrt{{\left(h^{\text{B\_base}}-0.45\right)}^{2}}}{\sigma^{\text{B\_base}}}}.$$ (17)

The current base height $h^{B\text{\_}base}$ can be obtained from the simulation environment, where 0.45 is the expected base height for the agent in a bipedal standing posture.

Based on the above, the total goal reward (Equation 18) for the bipedal state can be summarized as follows:

$$r^{\text{B}}=r^{\text{B\_gait}}+r^{\text{B\_v}}+r^{\text{base}}.$$ (18)

## 5 Experiments and results

We primarily address two questions: 1) To verify whether using the STA method can enhance learning efficiency and improve policy performance when the motion feature information in the expert dataset is incomplete. 2) To validate whether the adaptive skill command module can effectively balance the training of different skills.

We utilize the Isaac Gym simulation platform Makoviychuk et al. (2021) to parallelize 4,096 environments and employ the Proximal Policy Optimization (PPO) algorithm Schulman et al. (2017) for RL. Our experiments are conducted in two phases. Firstly, we use the Cassi method as a baseline to validate the effectiveness of STA. Subsequently, we evaluate our method, SMSL, in both simulation and on the real-world Solo8 robot Grimminger et al. (2020). As shown in Figure 3, our method was successfully deployed on the real robot. With a single policy, it not only learns non-similar skills but also autonomously generates transition motions. In the experiments, the robot demonstrated forward and backward movements in a quadrupedal stance, bipedal locomotion, and smooth transitions between quadrupedal and bipedal states. The configurations of the SMSL algorithm are shown in Table 2.

**TABLE 2:** Configuration.

Name	Value	Name	Value	Name	Value	Name	Value
$\omega^{I}$	0.02	$\omega^{G}$	1.0	$\omega^{\text{STA}}$	0.01	$\omega^{\text{Q\_gait}}/\omega^{\text{B\_gait}}$	5.0/4.0
$\omega^{\text{B\_base}}$	1.0	$\omega^{\text{fh}}$	1.0	$\omega^{v}$	1.0	$\sigma^{\text{fh}}$	0.025
$\sigma^{v}$	0.25	$\sigma^{\text{pitch}}$	1.0	$\sigma^{\text{base}}$	0.1	p	0.3

### 5.1 Versatility of STA modules

In the experiments, we used the vanilla Cassi method as a benchmark, excluding base velocity and joint velocity information from the imitation learning dataset because these velocity data are not directly observable. However, we retain the information of the quaternion and joint position, which can be obtained through simple measurement methods in the real world. We compare its performance before and after the incorporation of the STA method. The STA buffer is designed to store favorable features such as joint position, joint velocity, and base velocity from historical trajectories. This information will be used during the initialization phase of the environment. We design a series of agents that switch skills in the “trimesh” terrain within the Isaac Gym environment, and assess the performance of the policy by monitoring the survival rate of the agents. The experimental setup includes 500 agents starting from the same point, with each pair of skills forming a combination. Agents automatically switch skills after 500 timesteps, with a total timestep length of 1,000. If an agent resets during the experiment, we consider this as the agent ‘dying’.

**FIGURE 5:**
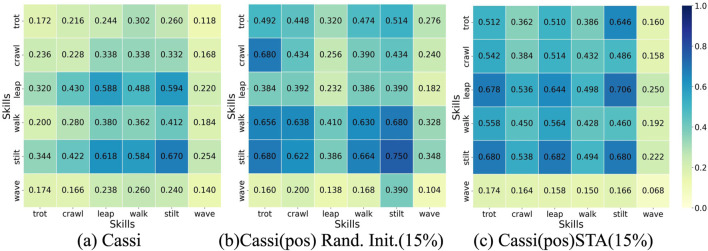
Agent Survival Rate Heatmap. The darker the shade of blue, the higher the survival rate for that pair of skills. Rand. Init. Stands for Random Initialization. **(a)** represents the original Cassi method. **(b)** represents the original Cassi method where the reference dataset retains only the robot’s joint position information, and the random initialization sampling probability is set to 15%. **(c)** represents the original Cassi method, where the reference dataset retains only the robot’s joint position information, and the STA method is introduced with a sampling probability set to 15%.

During the initialization phase of the Cassi algorithm, 85% of the samples are selected from the expert dataset, while 15% are randomly generated. Figure 5a displays the survival rates of agents in “trimesh” terrains using the Cassi method via a heatmap, where the expert dataset includes complete motion characteristics of the skills. Figure 5b (Cassi (pos)) and (c) (Cassi (STA)) both show scenarios after removing velocity information, with (c) specifically illustrating the application of the STA method in the with random initialization. Despite the removal of velocity information, the STA method still improves the survival rates of agents on untrained “trimesh” terrains, thereby enhancing the generalization of the strategy. Comparing the three methods, the survival rate for ‘wave’ is consistently low, mainly due to the skill’s intrinsic properties. The wave-like nature of its base motion trajectory increases the likelihood of contact with the ground in “trimesh” terrains, resulting in a lower survival rate. Despite some skill pairs showing slightly higher survival rates in Figure 5b, this may be due to the lack of a strict one-to-one correspondence between commands and skills, leading to additional training opportunities for some skills. Detailed analysis and data will be presented later in the text.

**FIGURE 6:**
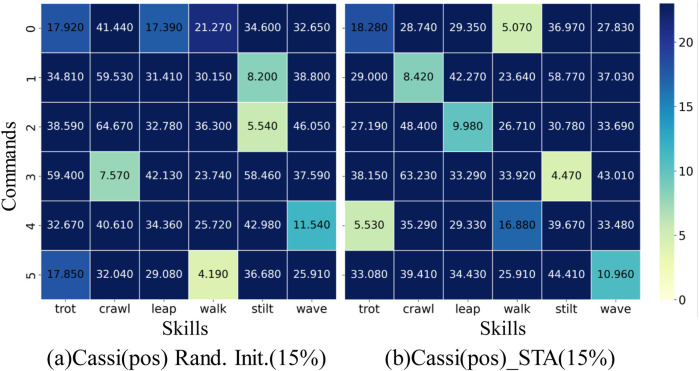
Skills-Commands Correspondence Diagram. The y-axis lists six commands, while the x-axis represents the skills in the expert dataset. The depth of the color indicates the level of similarity between the skills and commands; the darker the color, the lower the similarity. Ideally, each command should correspond to the lightest color block, indicating an exact match between the skill and the command. Similarly, each skill should also have only one light color block, ensuring a one-to-one correspondence between skills and commands. **(a)** represents the original Cassi method where the reference dataset retains only the robot’s joint position information, and the random initialization sampling probability is set to 15%. **(b)** represents the original Cassi method, where the reference dataset retains only the robot’s joint position information, and the STA method is introduced with a sampling probability set to 15%.

We extract state trajectories of six skills from the initial strategy of the Cassi algorithm and constructed a baseline dataset for comparative analysis. Using DTW technology, we calculate the distances between each skill trained by the Cassi (pos) method and the Cassi (STA) method and their corresponding skills in the baseline dataset. Ideally, each command should form a one-to-one correspondence with a specific skill. As shown in Figure 6a, when imitating the expert dataset, the mapping between skills and commands has errors due to the absence of some motion feature information. Specifically, in Figure 6a, command “0″ maps to two skills, “trot” and “leap,” while skill “stilt” corresponds to two different commands, “1″ and “2″ Theoretically, this could result in skill “stilt” receiving more training opportunities, and the data in the figure supports this view, showing it has the highest survival rate. Furthermore, Figure 6b displays the strategy trained by the Cassi (STA) method in the context of incomplete information from the expert dataset. Even under conditions of missing information, this method still manages to achieve a one-to-one correspondence between skills and commands. Additionally, the data in the figure indicates that the strategy trained by the Cassi (STA) method exhibits a relatively uniform distribution in terms of survival rates, meaning that each skill received balanced training opportunities.

In the final test, we assess the resilience of the Cassi (STA) method relative to the original Cassi method in the face of observational disturbances. In the simulation environment, we introduce random additive noise to the observations of joint positions and joint velocities, with ranges of [−0.3,0.3] and [−0.5,+0.5] , respectively. We then collect the joint state trajectories of the policies trained by both methods under six different skills, recording 500 timesteps for each skill. These trajectories are subsequently analyzed using DTW against the expert dataset. As shown in Tables 3, 4, even under varying levels of noise interference, the strategies trained using the Cassi (STA) method are closer to the expert dataset across most skills compared to those trained with the original Cassi method. This finding confirms the enhanced robustness of the Cassi (STA) approach.

**TABLE 3:** The DTW values for the Cassi with observational noise.

Noise Skills	Trot	Crawl	Leap	Walk	Stilt	Wave
[−0.3,+0.3]	14.03	**13.04**	**15.31**	12.24	13.37	16.61
[−0.5,+0.5]	21.74	**20.58**	22.77	19.38	20.94	25.38

**TABLE 4:** The DTW values for the Cassi (STA) with observational noise.

Noise Skills	Trot	Crawl	Leap	Walk	Stilt	Wave
[−0.3,+0.3]	**12.44**	14.15	16.10	**12.02**	**12.21**	**14.55**
[−0.5,+0.5]	**19.51**	20.61	**21.69**	**18.90**	**19.54**	**23.83**

### 5.2 Versatility of ACSM modules

**FIGURE 7:**
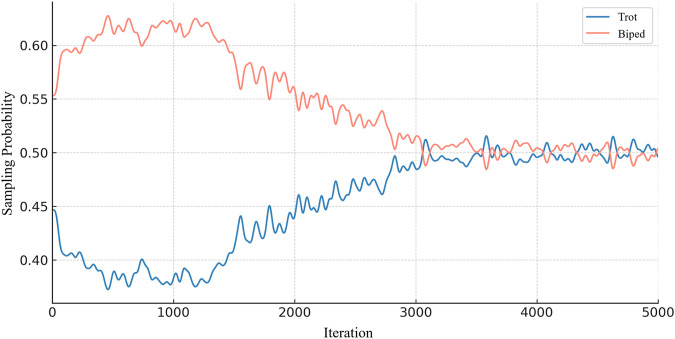
Variation in Skill Sampling Probabilities. The blue curve illustrates the sampling probability dynamics for the quadrupedal locomotion skill “Trot,” while the red curve corresponds to the bipedal locomotion skill “Biped.”

This experiment is designed to validate the effectiveness of the skill-adaptive module proposed in Section 4.2. Through meticulously designed experiments, we are able to observe and analyze the actual performance of the module during training, thereby evaluating its contribution to multi-skill learning. As shown in Figure 7, the experiment presents the variation in skill sampling probabilities over 5,000 iterations of training using the SMSL method. It is evident that at the outset of training, both skills are sampled with relatively high probabilities due to their low initial reward values. As the number of iterations increases, the quadrupedal trotting skill—owing to its inherent stability and relatively simple control requirements—becomes easier for the agent to learn and master, leading its sampling probability to quickly drop to approximately 50%. In contrast, the bipedal skill, characterized by greater control complexity and higher balance demands, is more challenging to learn; thus, in the early stages of training, its sampling probability is set relatively higher than that of the quadrupedal skill to ensure sufficient exploration and learning. Notably, to prevent the policy from exclusively focusing on the more difficult skill and suffering from catastrophic forgetting of the simpler one, a minimum sampling probability of 50% is maintained for each skill. This design guarantees that all skills receive adequate attention during the training process, thereby achieving comprehensive and balanced skill acquisition.

### 5.3 Ablation studies

**FIGURE 8:**
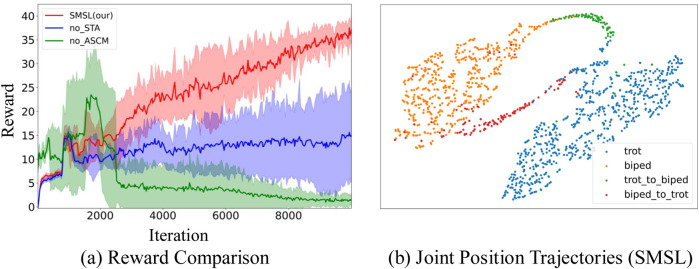
Performance display of the SMSL method. **(a)** Red represents the reward changes corresponding to each iteration of the SMSL method. Blue represents the reward changes with the STA method removed. Green represents the reward changes with the ASCM method removed. **(b)** Orange represents bipedal walking, blue represents quadrupedal walking, green represents transitions from quadrupedal to bipedal states, and red represents transitions from bipedal to quadrupedal states.

We conduct a detailed analysis of the roles of STA and ASCM within the SMSL framework (Figure 8a). By individually removing these methods from SMSL, using different seeds, and conducting training through over 10,000 iterations, we observe their impact on the effectiveness of agent learning. When the STA method is removed from the SMSL framework (represented by the blue curve), the agent struggles to effectively learn the strategy for transitioning between skills during the imitation learning process, due to the absence of transition actions between Non-similar skills in the expert dataset. This results in the agent tending towards blind exploration when faced with commands that require skill transitions, potentially leading to suboptimal local solutions. When the ASCM is removed (represented by the green curve), the agent, lacking a mechanism to balance training opportunities across skills, fails to adequately train more challenging skills such as bipedal walking. As a result, when the agent receives commands to perform bipedal walking, it may fail to execute them properly, exhibiting a collapse in strategy. The SMSL (Simultaneous Multi-Skill Learning) method effectively learns skills from a Non-similar expert dataset (represented by the red curve). Within this approach, the STA method extracts transitional actions between skills from historical states, facilitating effective exploration by the agent. Meanwhile, the ASCM balances the training opportunities across different skills, ensuring that the agent can comprehensively master all skills.

Figure 8b provides a T-SNE visualization that offers an intuitive representation, showcasing the joint trajectories for quadrupedal walking (trot, represented in blue) and bipedal walking (biped, represented in orange), as well as the transitions between them (represented in red and green). The joint trajectories for quadrupedal and bipedal walking are distinctly separated on the T-SNE plot, highlighted in blue and orange, respectively. This demonstrates that the SMSL method can effectively learn different motor skills from a Non-similar expert dataset. The red and green trajectories illustrate the transition actions between the two walking states, connecting the quadrupedal and bipedal trajectories. This indicates that the SMSL method not only learns individual motor skills but also generates seamless transitions between skills. In summary, the T-SNE plot in Figure 8b provides compelling visual evidence of the SMSL method’s powerful capability in multi-skill learning, particularly in handling Non-similar skills and their transitions.

## 6 Conclusion

We propose Seamless Multi-Skill Learning (SMSL) method, designed to enable a quadruped robot to learn Non-similar skills and their natural transitions from an incomplete, unlabeled expert dataset. This approach effectively simplifies the preparation phase of the expert dataset in multi-skill learning, reducing the complexity of the preparatory work. SMSL is based on the analysis of joint position information of robotic motion skills, utilizing this data for effective IL. A key advantage of this approach is its independence from the similarity between skills, it can achieve effective IL even when there is a significant difference between skills. This flexibility allows the agent to learn and adapt more comfortably when faced with a diverse set of skills. Additionally, SMSL places a strong emphasis on balancing the opportunities for skill training. Through this approach, we prevent the agent from falling into the trap of local optima during the learning process, ensuring global optimization. This balanced strategy helps the agent to master a comprehensive range of skills when faced with complex tasks, rather than being limited to a specific skill.

Experimental validation has demonstrated that our method significantly enhances the robustness of IL approaches. SMSL outperforms existing baseline methods in terms of the agent’s survival rate and the stability of skill-to-command mapping. These results indicate that SMSL has high practicality and effectiveness in real-world applications, especially in complex environments where an agent needs to flexibly switch between multiple skills.

SMSL significantly enhances the performance of quadruped robots in IL by simplifying the preparation process of expert datasets, enhancing the flexibility of skill learning, and balancing opportunities for skill training. These improvements not only increase the adaptability of agents in complex tasks but also bolster their robustness when facing new challenges.

Although our proposed method demonstrated significant potential in both simulated environments and experiments, we observed some challenges during the transition from simulation to real-world application. Specifically, the robot exhibited strong robustness in the quadrupedal state, while the bipedal state showed less stable movement. We conducted a detailed analysis of this phenomenon and identified several possible causes.

• **S**tructural Limitations: The robot model we used, Solo8, has a foot structure designed as a curved surface. In the bipedal motion state, this design results in degrees of freedom exceeding the number of actuators, thereby creating an underactuated motion. This underactuation makes controlling the robot in a bipedal state more complex and challenging to achieve precise motion controlLéziart (2022).

• Simulation and Reality Differences: Bipedal movement is more sensitive to discrepancies between simulated and real-world environments compared to quadrupedal movement. These differences may include sensor accuracy, environmental complexity, and the ways in which the robot interacts with its environment. These factors might be simplified or overlooked in simulations, but can significantly impact the robot’s movement in real-world applications. We refer to these as “compounding errors,” which accumulate between simulation and reality, leading to performance that may not meet expectations in actual environments.

Here are several main directions for our future work:

• Enhancing the robustness of control policy: Methods to enhance the robustness of robots in the real world can be divided into mechanical innovation and algorithmic innovation. Since mechanical methods are not the main focus of this thesis, we consider updates on the algorithmic side. To bridge the gap between real and simulated environments, a robust adversarial reinforcement learning method can be introduced. This method involves adversarial training with multiple agents, reinforcing the learning of the agents to compensate for the discrepancies between real and simulated environments Zhai et al. (2022).

• Algorithm Generality: Our aim is to explore a universal algorithmic framework that can be applied to various robotic platforms. The next step involves fine-tuning and training the algorithm for different robots.

• More skills, greater dissimilarit: The SMSL Method focuses on using a single policy to simulate and learn multiple skills, with the flexibility to switch between these skills seamlessly. This approach effectively addresses the potential decrease in robustness that may arise from multi-policy transitions. Moreover, the design of a single policy also somewhat reduces the burden on robotic systems in terms of storage and computation of strategy parameters. We will continue to leverage the advantages of this method to further optimize the algorithm, aiming to facilitate the learning and mastery of an even broader array of skills, including those with significant differences.

## Data Availability

The original contributions presented in the study are included in the article/Supplementary Material, further inquiries can be directed to the corresponding authors.
